# Special Issue: “Molecular Diagnosis and Treatments of Diabetes Mellitus”

**DOI:** 10.3390/ijms27010222

**Published:** 2025-12-25

**Authors:** Mihail Virgil Boldeanu

**Affiliations:** Department of Immunology, Faculty of Medicine, University of Medicine and Pharmacy of Craiova, 200349 Craiova, Romania; mihail.boldeanu@umfcv.ro

## 1. Introduction

Diabetes mellitus, particularly type 2 diabetes (T2DM), is increasingly being recognized as a systemic disorder that links metabolic dysregulation with immune activation and neurochemical imbalance. Beyond chronic hyperglycemia and dyslipidemia, individuals traverse a continuum from normoglycemia through prediabetes to overt T2DM, along which cardiometabolic risk diverges by phenotype rather than body mass alone, reflecting differences in adipose biology, hepatic lipid handling, autonomic tone, and low-grade inflammation [1,2,3]. Within these networks, neurotransmitters such as dopamine, norepinephrine, and serotonin exert essential regulatory functions, influencing insulin secretion, hepatic glucose production, adipose tissue metabolism, thermogenesis, and behavioral responses to nutrient availability [4,5,6]. Recent findings further indicate that dysregulation of these neurotransmitter systems may contribute to early variations in glycemic responses, body weight dynamics, and metabolic flexibility among individuals with prediabetes and new-onset T2DM [4,5,6].

The gut–brain axis represents another key integrative component linking nutrition, metabolism, immunity, and neural regulation. Alterations in gut microbiota composition, intestinal permeability, and microbial metabolites can affect incretin secretion, vagal signaling, bile acid pathways, and systemic inflammation, ultimately modulating glucose homeostasis and metabolic function [7,8]. Immune–enteric interactions further contribute to this bidirectional regulation, as crosstalk between intestinal immune cells and the enteric nervous system influences central metabolic control and systemic insulin sensitivity [9,10,11]. Disruptions of this axis have also been associated with changes in satiety, stress responses, mood symptoms, and the onset of diabetic neuropathy, highlighting the shared neuroimmune origins of metabolic and neurological complications in diabetes [9,10,11].

Low-grade chronic inflammation serves as a unifying mechanism linking metabolic dysfunction with neural and vascular injury. Activation of the nuclear factor kappa-light-chain-enhancer of activated B cells (NF-κB) and the nucleotide-binding oligomerization domain-like receptor family pyrin domain containing 3 (NLRP3) inflammasome contributes to β-cell stress, impaired insulin action, endothelial dysfunction, and neuroinflammation, thus promoting metabolic deterioration and amplifying the risk of diabetes-related complications [12,13]. In parallel, insulin resistance at the neuronal level, together with mitochondrial stress and oxidative injury, has been implicated in neurodegenerative processes observed in diabetes and metabolic syndrome, reinforcing the concept of a neurometabolic interface [14].

Diabetic neuropathy exemplifies the complexity of these intertwined mechanisms. Painful diabetic neuropathy results from the convergence of metabolic stress, microvascular ischemia, mitochondrial injury, and altered neurotransmitter signaling [14,15]. Established pathogenic pathways such as glycotoxicity and oxidative stress remain central, yet emerging evidence highlights the contributions of immune–neural crosstalk, central sensitization, and gut-derived inflammatory mediators in shaping neuropathic symptomatology and its early expression in prediabetes [9,15]. These overlapping influences help explain the marked heterogeneity of neuropathic pain phenotypes observed in early dysglycemia.

Recent research, including our own contributions, supports integrating metabolic, inflammatory, and neurochemical markers into precision phenotyping. We demonstrated that cardiometabolic clusters defined by obesity, insulin resistance, and glycoxidative stress display distinct biochemical signatures among individuals with prediabetes and new-onset T2DM [3]. Furthermore, we found that circulating neurotransmitters—including dopamine, norepinephrine, epinephrine, and serotonin—correlate with lipid parameters and metabolic indices, suggesting their potential utility as early biomarkers of metabolic dysfunction [2]. These findings position neurotransmitter and immune–metabolic pathways at the core of early diabetic pathophysiology and reinforce the need for mechanistic frameworks that bridge neurobiology, metabolism, and inflammation.

## 2. An Overview of Published Articles

This Editorial pertains to the Special Issue “Molecular Diagnosis and Treatments of Diabetes Mellitus.” This Special Issue of the *International Journal of Molecular Sciences* features 10 original research and review articles covering various aspects of diabetes pathophysiology, including neurotransmitter expression and genetic factors, immune–metabolic biomarkers, and innovative treatment approaches. The studies included discuss both clinical and experimental models, highlighting the complexity and multidimensional aspects of diabetes and its comorbidities.

The contributions can be categorized into four main thematic areas: (1) genetics and the molecular basis of diabetic complications, (2) metabolic regulation through diet and medications, (3) inflammation and biomarker identification, and (4) innovative or comprehensive therapeutic approaches.

### 2.1. Genetic and Molecular Signatures in Diabetic Neuropathy

The review by Noémi Hajdú (Contribution 1) summarizes current evidence on genetic susceptibility to diabetic neuropathy. The authors highlight how specific polymorphisms in genes such as the angiotensin-converting enzyme (ACE), glutathione S-transferase-mu (GSTM1), methylenetetrahydrofolate reductase (MTHFR), apolipoprotein E (APOE), and the transient receptor potential cation channel subfamily A, member 1 (TRPA1) influence both the risk and symptoms of neuropathy—sometimes regardless of glycemic control. Notably, the paper emphasizes underexplored interactions between hyperglycemia-driven metabolic pathways and genetic variations, encouraging future research on gene–environment interactions and personalized preventive strategies.

Complementing this, the review authored by Natasha Ivanova (Contribution 2) offers a detailed overview of rodent models of painful diabetic neuropathy (PDN), emphasizing dysregulated calcium homeostasis and the therapeutic potential of inhibiting Na^+^/Ca^2+^ exchange. Their original research on KB-R7943 identifies this agent as a potential modulator of nociception and depressive symptoms linked to PDN, providing a new pharmacological approach based on ion regulation.

### 2.2. Dietary and Pharmacologic Modulation of Insulin Sensitivity and Neuroprotection

Two interventional studies included in this Special Issue explore dietary and medication strategies for improving metabolic control. The article authored by Neda Rajamand Ekberg (Contribution 3) describes a six-month, non-randomized controlled trial that assessed the effectiveness of the 5:2 intermittent fasting diet in individuals with and without T2DM. Their findings revealed sustained reductions in the Homeostatic Model Assessment for Insulin Resistance (HOMA-IR), C-peptide, insulin-like growth factor binding protein-1 (IGFBP-1), and visceral fat, resulting in long-term improvements in insulin sensitivity and liver health markers.

In a separate preclinical study, Lin-Li Chang (Contribution 4) examined the effects of cilostazol in streptozotocin-induced diabetic rats. This phosphodiesterase inhibitor improved motor function, preserved Schwann cell integrity, and decreased markers of neuropathic damage. Notably, cilostazol offered neuroprotective benefits independent of glycemic control, indicating its potential as an additional therapy for diabetic peripheral neuropathy (DPN).

### 2.3. Inflammation and Biomarkers: From Adipose Signaling to Clinical Predictors

Several studies in this special issue emphasize the crucial role of inflammation in the development and progression of diabetes. Research by Roxana-Viorela Ahrițculesei (Contribution 5) examined various inflammatory biomarkers (pentraxin 3 (PTX3), high-sensitivity C-reactive protein (hs-CRP), interleukin-6 (IL-6), tumor necrosis factor-alpha (TNF-α)) and new indices (the mean corpuscular volume (MCV) to lymphocyte ratio (MCVL) and the cumulative inflammatory index (IIC)) in patients with prediabetes and newly diagnosed T2DM. PTX3 and MCVL demonstrated promising diagnostic accuracy and significant correlations with obesity-related measures, offering accessible tools for early risk assessment.

Anton Hellberg (Contribution 6) studied adipose tissue gene expression in overweight women with polycystic ovary syndrome (PCOS), identifying GSTM5, ANLN, and H3C2 as predictors of successful weight loss through behavioral intervention. These findings improve our understanding of the molecular link between adipose dysfunction, metabolic inflexibility, and therapeutic response.

From a vascular and neuropathic perspective, the article by Marcell Hernyák (Contribution 7) identifies kallistatin as a potential biomarker for therapeutic response in DSPN patients treated with alpha-lipoic acid. Reductions in kallistatin correlated with improvements in endothelial dysfunction and inflammatory cytokines, opening up new possibilities for targeted antioxidant monitoring.

### 2.4. Integrative and Immunomodulatory Therapies: From TCM to Autoantigen Vaccines

In their examination of integrative medicine, Jiawen Huang et al. (Contribution 8) reviewed the role of traditional Chinese medicine (TCM) in regulating nuclear receptors, including the farnesoid X receptor (FXR), liver X receptor (LXR), and peroxisome proliferator-activated receptors (PPARs), in diabetes. The authors offer a compelling synthesis of how TCM formulations affect bile acid signaling, glucose regulation, and inflammatory pathways—providing a systems biology explanation for their clinical effectiveness.

In a forward-looking pilot trial, Rosaura Casas (Contribution 9) assessed the effects of redosing intralymphatic glutamic acid decarboxylase (GAD)-alum immunotherapy in patients with type 1 diabetes (T1D) who carry the human leukocyte antigen DR3-DQ2 (HLA DR3-DQ2) haplotype. The results showed stability in C-peptide and glycosylated hemoglobin A1c (HbA1c) over 12 months, suggesting that repeated antigen-specific immunotherapy may help extend β-cell preservation in genetically defined subgroups.

To close the immunological loop, Henrik Toft-Hansen’s work (Contribution 10) systematically examined anti-insulin antibodies in T1D and T2D patients. Despite their high prevalence, these antibodies did not show a significant association with glycemic control, suggesting limited clinical relevance for current insulin therapy regimens.

Together, these ten contributions span from mechanistic to translational science, covering genetics, immunology, nutrition, vascular biology, and neuroendocrinology. They highlight the importance of a multidimensional view of diabetes—one that integrates metabolic, inflammatory, and neuronal pathways.

As Guest Editor, I sincerely thank all the authors, reviewers, and editorial staff for their dedication to advancing this important field. I hope this collection inspires further research into integrative diagnostics and personalized treatments for diabetes, neuropathy, and related metabolic disorders.

## 3. Conclusions

The studies presented in this Special Issue highlight the increasing convergence of molecular pathophysiology, biomarker discovery, and translational therapeutics in diabetes. The integration of genomic, neurochemical, and inflammatory profiling is gradually transforming how we understand disease heterogeneity, uncovering disease-specific phenotypes that go beyond traditional glycemic classifications. By combining basic and clinical research—from neurotransmitter biology and immune signaling to drug development and lifestyle strategies—this Special Issue offers a thorough overview of the molecular underpinnings behind both metabolic and neurological complications of diabetes.

Looking ahead, the field is advancing toward precision approaches that link molecular data with personalized treatments. Progress in multiomics, high-throughput metabolomics, and neuroimaging is expected to identify actionable biomarkers that predict the transition from prediabetes to T2DM, as well as the development of neuropathy, retinopathy, and cognitive decline. Efforts to translate research by integrating gut–brain–metabolic axis studies with neuroendocrine and immune pathways will likely yield new therapeutic options that target not only blood sugar control but also inflammation and neuronal issues. Using the multidimensional perspective presented by the authors in this Special Issue, diabetes appears as a systemic, network-based disease—one that necessitates comprehensive diagnostics and targeted, mechanism-based treatment strategies.
